# Preliminary seroprevalence study of zoonotic abortigenic agents in the abortion inexperienced sheep population in the Northern Cyprus

**DOI:** 10.1007/s11259-025-10790-0

**Published:** 2025-06-10

**Authors:** Hasan Baloğlu, Hatice Esra Çolakoğlu, Nazlı Senem Cam

**Affiliations:** 1 NC Ministry of Agriculture and Natural Resources, Lefkoşa, Northern Cyprus; 2 Faculty of Veterinary Medicine, Department of Gynecology and Obstetrics, Ankara University, Ankara, 06110 Turkey; 3 Graduate School of Health Sciences, Ankara University, Ankara, 06110 Turkey

**Keywords:** Abortion, Northern Cyprus, Seroprevalance, Sheep

## Abstract

There is no detailed and comprehensive study to determine the seroprevalence of zoonotic infections that cause abortion in sheep in Northern Cyprus. The study was conducted in the 3 districts with the highest sheep population in Northern Cyprus. This cross-sectional study aimed to serologically determine *Brucella abortus*,* Brucella melitensis*,* Toxoplasma gondii*, *Coxiella burnetii* and *Chlamydia abortus*, which are the agents of zoonotic abortion in sheep and to investigate the seroprevalence rate in these 3 regions. A total of 450 serum samples were collected from 45 farms located in the districts of Lefkoşa, Vadili and Ziyamet, where the sheep population is highest in Northern Cyprus. Serum samples were taken from 10 sheep in 15 farms, in each of the three districts for a total of 450 samples, and exposure to *Brucella spp*., *Toxoplasma gondii*, *Coxiella burnetii* and *Chlamydia abortus* were investigated. Brucellosis was found negative in all serum samples with the complement fixation test. With the ELISA test, 96 (21.33%) sheep were detected as *Toxoplasma gondii* positive, 175 (38.88%) sheep as *Coxiella burnetii* positive and 14 (3.11%) sheep sera were detected as *Chlamydia abortus* positive. With the eradication studies implemented in Northern Cyprus, it was determined that Brucellosis was no longer a problem in this region, that *T. gondii*, *C. burnetii* and *C. abortus* pathogens were detected in Northern Cyprus, and that the zoonotic abortion problem in this region could be reduced by taking biosecurity measures.

## Introduction

The livestock sector, which has been developing on the island of Cyprus in recent years, makes serious contributions to the country’s economy. According to the 2024 data of the Veterinary Department Directorate, there are approximately 350,602 breeding ovine (goat and sheep) animals in 4055 farms. The number of animals on farms in the region varies between 50 and 1000. However, in intensive and semi-intensive farms, breeding is generally carried out in flocks of 200–300 heads. The most intensive breeding is the various crossbreed of the Chios and Awassi races.

In order to increase the meat and milk yield of small ruminants to the desired levels, it is necessary to maintain optimal fertility. Abortion in sheep and goats significantly affects herd productivity. Abortion cases that spread throughout the herds and, thus, infertility problems cause significant economic losses and also put public health at risk due to zoonotic agents. Abortions, which can be classified as infectious and non-infectious, can be encountered at any period during the pregnancy of small ruminants. The diagnosis rate of the causes of abortion varies between 5% and 50% in the world. Although the exact rate of infectious abortions is unknown, 90% of the cases in which the cause is determined are infectious (Givens et al. 2008; Guesmi et al. 2023). Although the causative agents of abortion in small ruminants are mainly bacterial, viral, parasitic and fungal infections are also encountered. Abortion cases are caused mainly by Brucellosis, Campylobacteriosis, Salmonellosis, Chlamydiosis, Leptospirosis, Listeriosis, Bluetongue, Border Disease and Toxoplasmosis (Guesmi et al. 2023; Nogarol et al. 2024).

The economic losses caused by abortions in small ruminants and the threat they pose to public health are known, but there is no study on the classification of these infectious diseases in the Northern Cyprus. In this context, this cross-sectional study aims to determine the seroprevalence of *Brucella spp.* (*Brucella abortus*,* Brucella melitensis)*,* Toxoplasma gondii*, *Coxiella burnetii* and *Chlamydia abortus* in the sheep population in the Northern Cyprus. With the data obtained from the study, it is aimed to contribute to the national economy and public health with the necessary control and protection methods against zoonotic diseases that cause abortion in sheep in the country where small ruminant breeding is widespread. In addition, since there is no previous research on four infectious diseases in the Northern Cyprus, the results will likely contribute to the country’s data.

## Materials and methods

### The study area and sampling

The cross-sectional study was carried out between January and May 2024 in 45 farms located in Nicosia, Vadili and Ziyamet, which are the three districts with the densest sheep populations among the eight districts of the Northern Cyprus. Ten female sheep of various breeds (Chios, Ivesi, Assaf) selected by random sampling in 45 different farms in Nicosia, Vadili and Ziyamet, 15 farms in each district, were used as study material. Samples were taken from herds of 200–300 animals. Within the scope of the study, blood samples were taken from ewes between the ages of 1–10 years, without a history of abortion and any clinical symptoms during sampling. Permission was obtained from the Near East University Local Ethics Committee for Animal Experiments and Northern Cyprus Ministry of Agriculture and Natural Resources (letter dated 23.07.2023 and numbered 162). The number of samples studied was determined using the power analysis method. In the seroprevalence study, the sample size was calculated, as stated by Naing et al. (2022). The sample size was calculated with the assumption that the prevalence would be 50%, and the sample size was calculated as at least 385 animals.

### Analysis of samples

A total of 450 whole blood samples (5 ml), 10 from each herd, were collected in sterile Vacutainer tubes without anticoagulant for serological tests. Blood samples were immediately cooled to 4 °C and sent to the laboratory. Serum samples obtained by centrifuging at 3,000 × g for 10 min were stored at −20 °C until analysis.

The presence of Brucella spp. *(B. abortus*,* B. melitensis)* antibodies was investigated with a commercial kit, “IDEXX Rose Bengal Antigen”. Rose Bengal Plate Test (RBPT) was first performed as a screening test for Brucellosis. Sera showing seropositive reactions as a result of RBPT were confirmed by enzyme-linked immunosorbent assay (ELISA) (Svanovır^®^Brucella Ab-C-ELISA) or complement fixation test (CFT) methods (Alton et al. 1988; OIE 2009). When evaluating the test results, samples showing the presence of agglutination (even mild) according to the user instructions were accepted as positive samples.

The presence of *C. abortus* antibodies was investigated with a commercial ELISA kit (ID.vet, ID Screen *C. abortus* Indirect Multi-Species). Samples, positive and negative controls were tested according to the kit procedure and absorbance values (OD) were measured at 450 nm on an ELISA reader. According to the test procedure, the S/P ratio of each blood serum sample was calculated as %. The S/P % of the samples were evaluated as > 50% = positive, ≤ 50% to ≥ 10% = suspect, and < 10% = negative.

The presence of *T. gondii* antibodies was investigated with a commercial ELISA kit (ID.vet, ID Screen Toxoplasmosis Indirect Multi-Species). Samples, positive and negative controls were tested according to the kit procedure and absorbance values (OD) were measured at 450 nm on an ELISA reader. According to the test procedure, the S/P ratio of each blood serum sample was calculated as %, and the % S/P ratios of the samples were evaluated as S/P % ≤ 40% Negative, 40% < S/P % ≤ 50% Suspect and S/P % ≥ 50% Positive.

The presence of *C. burnetii* antibodies was investigated with a commercial ELISA kit (ID.vet, ID Screen Q Fever Indirect Multi-Species). Samples, positive and negative controls were tested according to the kit procedure and absorbance values (OD) were measured at 450 nm on an ELISA reader. According to the test procedure, the S/P ratio of each blood serum sample was calculated as % S/P and the % S/P ratios of the samples were evaluated as % S/*P* ≤ 40% Negative, 40% < S/P % ≤ 50% Suspect and 50% < S/P % ≤ 80% Positive.

### Data analysis

Descriptive statistics were calculated as “Frequency-Percentage” and shown in tabular form. The evaluation of different regional characteristics recorded in the study in terms of prevalence was determined by the chi-square test. *P* < 0.05 was accepted as the statistical significance level, and statistical analyses were evaluated using the Stata 15.1 statistical program (Naing et al. 2022).

## Results

The results of the tests performed to determine Brucella spp., *T. gondii*, *C. burnetii* and *C. abortus* antibodies in the serum samples of the sheep sampled within the scope of the study and their distribution according to the districts are given in Table 1. One (1) of 450 RBPT samples for Brucellosis showed positive results, but the presence of antibodies could not be detected in the confirmation test performed with the complement fixation test, which has a higher specificity. This was considered a non-specific titer increase and was not taken into account. As a result of the study, *Brucellosis* was not detected in any of the 450 samples, and the seroprevalence of the agent was determined to be 0%. Fourteen (3.11%) of the 450 samples were positive for *C. abortus* and at least one animal was positive in 8 of the 45 farms (17.77%). The farm-based seroprevalence values in the three regions (Lefkoşa, Ziyamet, Vadili) were determined as 40%, 0%, and 13,33%, respectively. Of the 450 samples studied, 96 (21.33%) were positive for *T. gondii.* Seroprevalence values on farm basis in Lefkoşa, Ziyamet and Vadili were determined as as 46,66%, 80%, and 66,66%, respectively. At least one animal was positive in 29 out of 45 farms (64.44%). 175 (38.88%) out of 450 samples were evaluated as *Coxiella burnetii* positive, and at least one animal was positive in 40 out of 45 farms (88.88%). The farm-based seroprevalence values ​​in the three regions (Lefkoşa, Ziyamet, Vadili) were determined as 93,33%, 93,33%, and 80%, respectively.

**Table 1:** Seroprevalence rates of the abortion causing agents by region

Infections		*Brucella* spp. (*B. abortus*, *B. melitensis*)	*C. abortu*s*	*T. gondii* ^+^	*C. burnetii* ^≠^
Seroprevalence rate % (no.of positive ewes)		% (*N*)	% (*N*)	% (*N*)	% (*N*)
**Locality**	**Lefkoşa**	0%	7,33% (11)^a^	20,67% (31)	39,33% (59)
	**Ziyamet**	0%	0%^b^	20,67% (31)	37,33% (56)
	**Vadili**	0%	2% (3)^b^	22,67% (34)	40% (60)
**P**			*P* = 0,*001*	*P* = 0,*888*	*P* = 0,*821*

* %95 CI: Lefkoşa 3,16 − 11,5; Vadili 0–4,24

^+^ %95 CI: Lefkoşa 17,36 − 23,98; Ziyamet 17,36 − 23,98; Vadili 19,25–26,09

^≠^ %95 CI: Lefkoşa 35,34–43,32; Ziyamet 33,38–41,28; Vadili 36–44

## Discussion

In sheep-goat breeding, in order to increase meat and milk yield to the target levels, it is necessary to maintain reproductive efficiency optimally. The most important problem that disrupts reproductive efficiency is abortion. Although the effect of individual abortion and infertility is small, the problem of generalized abortion in herds causes serious economic losses, and zoonotic agents also negatively affect public health. In order to prevent abortion and related economic and health problems, it is very important to determine the common etiologic agent in the region (Guner et al. 2016).

Abortions are one of the most important economic problems of sheep farms in Northern Cyprus (2024 data of the Veterinary Department Directorate). There is no detailed and comprehensive study on infectious agents causing abortions in sheep and goats in Northern Cyprus. In the presented study, the seroprevalence of *Brucella* spp. was determined as 0%. In 2022, in a prevalence study conducted in Ethiopia, the prevalence of Brucellosis was determined to be 5.4% (Sorsa et al. 2022). In 2021, Gompo et al. reported a 15% Brucellosis prevalence in sheep in Nepal, Guesmi et al. (2023) reported 16.1% in Tunisia, and Meletis et al. (2024) reported an 18% Brucellosis prevalence in Iran. Higher Brucellosis prevalence was determined in previous studies conducted in different regions. Within the scope of the Brucellosis eradication project in the Northern Cyprus, the prevalence of Brucellosis showed a decreasing momentum with 10.2% in 2016–2017, 6.17% in 2018–2020, 5.09% in 2021, 1.58% in 2022, 1.58% in 2022 and 0.23% in 2023 (NC Ministry of Agriculture and Natural Resources Directorate of Veterinary Department 2023 Annual Report). It is seen that the eradication efforts and measures taken in the region were successful and Brucellosis was eradicated and therefore could not be detected serologically in the study.

In the present study, the animal seroprevalence of *C. abortus* was found to be 3.11%, and it was determined to have the lowest seroprevalence of the remaining three pathogens that were studied. It is the most frequently isolated bacterium in abortion cases in many regions where sheep farming is developed, such as North America, Australia, New Zealand and Europe. Since Brucellosis can be eradicated in such countries, *C. abortus* seems to be the major agent for abortions (Guner et al. 2016). Nogarol et al. (2024) determined the herd prevalence of *C. abortus* to be 56.6% in a study conducted with a total of 3045 sheep and goats in Italy. Iraninezhad et al. (2020) determined the prevalence of *C. abortus* in sheep and goat population as 9.7% in their study in Iran. Similarly, the prevalence of *C. abortus* in sheep was determined as 10.9% in Saudi Arabia (Fayez et al. 2021) and 13.77% in Egypt (Selim et al. 2021) and was found to have a higher prevalence than the present study. The prevalence of *C. abortus* was determined to be 58% in Tunisia in 2020, which is considerably higher than the presented study (Mamlouk et al. 2020). In Italy, 56.6% prevalence was determined on a herd basis (Nogarol et al. 2024). It is thought that the prevalence of *C. abortus* may differ due to regional differences as well as the diagnostic method used, sampling methods and times, infection rate, vaccination status and training of animal breeders. It is also thought that the lower seroprevalence than other countries may be due to the fact that asymptomatic sheep with no history of abortion were studied. In addition, this low seroprevalence suggests that breeders control this disease more effectively in their herds than in other countries.

In the present study, *T. gondii* was the second most frequently detected agent, with 96 positive samples and 21.33% individual seroprevalence. There are many studies in which the prevalence of *T. gondii* in sheep was determined. The prevalence of *T. gondii* was 33.97% in Italy (Cenci-Goga et al. 2013), 17.78% in China (Jia et al. 2023), 57.1% in Greece in 2013 (Diakoua et al. 2013), 36.8% in Poland in 2018 (Moskwa et al. 2018), and 51.76% in Egypt in 2017 (Ibrahim et al. 2017). Guesmi et al. investigated the seroprevalence of *T. gondii* by ELISA in 793 sheep in Tunisia in 2023 and found a prevalence of 19.7%. Subedi et al. (2018) determined the prevalence of *T. gondii* in sheep to be 36.17% in Nepal. In a study conducted in the Northern Cyprus in 1999, the prevalence was found to be 52.11%, which is quite high (Nalbantoglu et al. 1999), and although it has decreased with the preventive measures taken over the years, its seriousness continues. Due to its zoonotic and economic importance, the prevalence determined in the Northern Cyprus is not to be underestimated, and it is determined that it should be taken under control with prevention and control measures.

In the current study, C. burnetii was the agent with the highest seroprevalence with 175 positive samples and 38.88% seroprevalence. *C. burnetii* ranks first as the zoonotic agent in Nicosia, Vadili and Ziyamet districts in Northern Cyprus. In a study conducted in Egypt, the prevalence was found to be 25.5%. In a study conducted in Greece in 2017, and in a study conducted in Jordan, the prevalence of *C. burnetii* was 27%. In a study conducted in Tunisia in 2023, *C. burnetii* seroprevalence was determined as 17.2% by the ELISA method (Guesmi et al. 2023). In a study conducted in Iran to determine the prevalence of abortion agents in sheep, the most intensively isolated agent was *C. burnetii*, with 22.7% and ranked first, similar to the prevalence results in Northern Cyprus (Esmaeili et al. 2022). *Chlamydia abortus* (12.3%) was isolated in the second place and *B. melitensis* (10.4%) in the third place (Esmaeili et al. 2022). In a study conducted in Italy in 2024, the presence of *Coxiella burnetii* antibodies in serum samples was determined to be 53.6–77.0% (Blanda et al. 2024). The prevalence of *C. burnetii* was 14.5% in the USA (Welch et al. 2024). In previous studies conducted in different regions (USA, Tunusia, Greece), the prevalence of *C. burnetii* was much lower than in the present study. The high prevalence of *C. burnetii*, a zoonotic abortion agent, indicates that it is a significant problem for Northern Cyprus and a significant threat to public and animal health in these regions. It is thought that the high prevalence of the agent, which is reported to be excreted by sheep with their vaginal discharge or feces, may have been caused by insufficient implementation of necessary biosecurity measures. More studies and special attention to control programs are needed to counter *C. burnetii*, which is zoonotic and has a high prevalence.

The fact that sheep with a history of abortion were not included as a separate group constitutes the limitations of the study. In addition, this study was limited to zoonotic abortion agents. Including non-zoonotic abortion agents in the study would be more beneficial in terms of enriching and expanding the finding.

## Conclusion

In conclusion, this is the first study on the seroprevalence of *B. abortus*,* B. melitensis*,* T. gondii*, *C. burnetii* and *C. abortus* in sheep in Northern Cyprus. It was determined that the zoonotic abortion agent with the highest seroprevalence is *C. burnetii* sheep in Northern Cyprus, *T. gondii* and *C. abortus* agents were in the second and third place, and Brucellosis was no longer a problem for the mentioned regions as a result of eradication measures. Considering the importance of zoonotic diseases, raising awareness among farmers and informing them about vaccination is of strategic importance. It was concluded that the data obtained in the study enabled the determination of the seroprevalence of zoonotic abortion agents in the Northern Cyprus sheep, that success can be achieved in the problem of abortion with control and eradication practices for these factors and that the data can make an important contribution to studies on abortion.

## Data Availability

No datasets were generated or analysed during the current study.
